# Inference on *P*(*X* < *Y*) in *Bivariate Lomax* model based on progressive type II censoring

**DOI:** 10.1371/journal.pone.0267981

**Published:** 2022-05-12

**Authors:** Amal Helu, Hani Samawi

**Affiliations:** 1 Department of Mathematics, The University of Jordan, Amman, Jordan; 2 Jiann-Ping Hsu College of Public Health, Georgia Southern University, Georgia Southern, Georgia, United States of America; Arizona State University, UNITED STATES

## Abstract

This article considers the estimation of the stress-strength reliability parameter, *θ* = *P*(*X* < *Y*), when both the stress (*X*) and the strength (*Y*) are dependent random variables from a Bivariate Lomax distribution based on a progressive type II censored sample. The maximum likelihood, the method of moments and the *Bayes* estimators are all derived. Bayesian estimators are obtained for both symmetric and asymmetric loss functions, via squared error and *Linex* loss functions, respectively. Since there is no closed form for the Bayes estimators, Lindley’s approximation is utilized to derive the Bayes estimators under these loss functions. An extensive simulation study is conducted to gauge the performance of the proposed estimators based on three criteria, namely, relative bias, mean squared error, and Pitman nearness probability. A real data application is provided to illustrate the performance of our proposed estimators through bootstrap analysis.

## 1. Introduction

It has always been the main objective of manufacturers to provide reliable products. For their products to remain desired and thus profitable, they strive to create high-quality and long-lasting products. In order to accomplish this, it is necessary to know the failure time distributions of products that are obtained by performing life testing experiments on them before they are released into the market.

In reliability studies, a sample of size *n* is subjected to a test for observing their failure times. The data recorded is then used to establish a time-to-failure distribution. This may be impractical, costly and time consuming since the experimenter may need to terminate the study before recording the failure times for all the subjects under consideration due to time constraints and facilities restrictions. In addition, some functioning tests’ subjects may need to be removed so they can be used in another test or to collect degradation related information about failure time data which is typically the case when the test subjects are expensive such as medical equipment. Moreover, in some cases, the failure is planned and predicted, but it does not occur due to operator flaw, equipment malfunction, test irregularity, etc. Samples that result from such situations are called censored samples.

The two most common types of censoring schemes in the literature are type-I and type-II censoring schemes, in which the test ceases at a pre-specified time, or after a predetermined number of failures. However, these censoring schemes do not allow intermediate removal of active units during the experiment other than the final termination point. Therefore, the focus in the last few years has been on progressive censoring.

Progressive type-II censoring is a generalization of type-II censoring. It allows researchers to remove subjects before the final termination point if certain circumstances arise, such as losing contact with the subjects. Under this type of censoring, *n* independent items are placed simultaneously on a life testing experiment, and only *m* (< *n*) failures are completely observed. The censoring occurs progressively in *m* stages as follows: When the first failure is observed, a random sample of size *R*_1_ is immediately drawn and removed from the (*n* − 1) survivals, hence, leaving *n* − 1 − *R*_1_ survival items. Then after the failure of the second item, the sample becomes *n* − 2 − *R*_1_, in which another sample of size *R*_2_ is randomly selected and removed from the remaining survival units. Continue with this process until *m* failures are observed, and all the remaining *n* − *m* − *R*_1_ − ⋯ −*R*_*m*−1_( = *R*_*m*_) surviving units are removed from the experiment. It is assumed that the lifetimes of these *n* units are independent and identically distributed with a common distribution function **F**. Moreover, *n*, *m*, and the censoring scheme **R** = (*R*_1_, *R*_2_, …, *R*_*m*_) are all pre-specified. Note that if *R*_1_ = *R*_2_ = ⋯ = *R*_*m*−1_ = 0, then *R*_*m*_ = *n*−*m* which corresponds to type-II censoring. If *R*_1_ = *R*_2_ = ⋯ = *R*_*m*_ = 0, then *m* = *n* which represents the complete data set. For a comprehensive literature review on progressive censoring, readers may refer to [1].

Considerable attention has been directed towards progressive type II censoring. This is largely due to the availability of the high-speed computing resources, which makes it feasible for simulation studies as well as a practical method of gathering lifetime data for both researchers and practitioners [2].

A vast number of researchers dedicated so much of their work to study the stress-strength model. [3] provided an interesting connection between the stress-strength empirical estimate and the classical Man-Whitney statistic. More works have followed to provide point and interval estimation of *θ* using different approaches. For example [4] provided a comprehensive review of the development of the stress-strength reliability and its applications until the year 2003. Recently, [5] studied the estimation of *θ* when *X* and *Y* are two independent generalized Pareto distributions with different parameters. [6] studied the reliability of the stress-strength model when *X* and *Y* are independent Poisson random variables. Whereas, [7] considered the problem of estimating *θ* when *X* and *Y* are distributed as Lindley with different shape parameters. [8] derived a point and interval estimation of *θ* using maximum likelihood, parametric and nonparametric bootstrap methods when *X* and *Y* are independent power Lindley random variables. [9] extended the work of [8] and developed a *Bayesian* inference on *θ*. In addition, [10] derived asymptotic confidence intervals for *θ* when *X* and *Y* are two independent generalized Pareto random variables with the same scale parameter.

However, all aforementioned results are based on the assumption that the strength (*X*) and the stress (*Y*) are independent random variables. Still little attention has been paid to the actual situation in which *X* and *Y* are dependent random variables. Recently, different estimators of *θ* have been proposed assuming several bivariate distribution families of (*X*, *Y*) for describing the joint behavior of strength and stress variables. Among others, [11] assumed that *X* and *Y* are marginally distributed as a skewed scale mixture of normal distributions and constructed the corresponding joint distribution using a Gaussian copula. [12] constructed the joint distribution of (*X*, *Y*) using a Farlie-Gumbel-Morgenstern copula. [13] derived *Bayesian* inference of *θ* when strength and stress are dependent random variables with a bivariate Rayleigh distribution. [14] considered the problem of estimating *θ* when *X* and *Y* are dependent random variables with a bivariate underlying distribution using kernel estimation and bivariate ranked set sampling. Recently, [15] investigated the asymptotic properties of the kernel density estimator based on progressive type II censoring and their application to hazard function estimation. [16] used classical and Bayesian estimation methods to derive estimates of *θ* when *X* and *Y* are dependent random variables from *Bivariate Lomax* distribution. [17] discussed the kernel based estimation of *θ* when *X* and *Y* are dependent random variables under bivariate progressive type II censored sample and provided asymptotic properties of the kernel estimators of *θ* based on progressive type-II censoring.

Little work has been done to study the reliability in case of bivariate random variables, and as far as we know, none is done to study the reliability in case of bivariate data based on progressive type II censoring.

The article describes the estimation of the reliability *θ* = *P*(*X* < *Y*) when *X* and *Y* are dependent random variables based on progressively type II censored samples from the *Bivariate Lomax* distribution using classical and *Bayesian* approaches. For the classical approach we propose using the method of moments (*MOM*) and the Maximum likelihood estimators (*MLE*). While for the *Bayesian* approach, we derive the estimates based on symmetric and asymmetric loss functions. It is observed that the *Bayes* estimates cannot be obtained in explicit form, so instead of using numerical techniques, approximation method such as Lindley’s approximation is applied. To compare the performance of the proposed estimators we used real life data. In addition, we used the bootstrap approach to calculate the bootstrap estimates, standard error, and lower and upper confidence interval limits.

The layout of this paper is as follows. In Section 2, we briefly describe the *Bivariate Lomax* model. The classical analysis is provided in Section 3. In Section 4, we discuss the *Bayesian* inference. Simulation studies which are conducted to assess the accuracy of the proposed methods are presented in Section 5. In Section 6, the analysis of real data set is presented for illustrative purposes. Finally, conclusions are presented in Section 7.

## 2. *Bivariate Lomax* model

This model was proposed by [18], considering a two component system where the component lifetimes *X* and *Y* are conditionally independently exponentially distributed with failure rates *η*λ_1_ and *η*λ_2_ respectively, where the parameter *η* represents the environment effect and λ_1_ and λ_2_ are the original failure rates. Then,

$$\begin{array}{c} f\left(x,y|\eta \right)=\int \eta \lambda_{1}e^{-\eta \lambda_{1}x}\eta \lambda_{2}e^{-\eta \lambda_{2}y}dG\left(\eta \right), \end{array}$$ (1)

where, *G*(*η*) is the cumulative distribution function (*cdf*) of the environment parameter *η*. In order to find the unconditional distribution of (*X*, *Y*), we assign the gamma distribution *g*_*η*_(*c*, *b*) as the distribution of *η*, with probability density function (*pdf*)

$$\begin{array}{c} g_{\eta }\left(c,b\right)=\frac{b^{c}\eta^{c-1}e^{-\eta b}}{\Gamma (c)},\;\;\;\eta ,c,b>0. \end{array}$$ (2)

Therefore, the joint density function of (*X*, *Y*) is given by

$$\begin{array}{ccc} f_{\left(X,Y\right)}\left(x,y\right) & = & \int f\left(x,y|\eta \right)g_{\eta }\left(c,b\right)d\eta \\ & = & \frac{\lambda_{1}\lambda_{2}b^{c}c\left(c+1\right)}{{\left(b+\lambda_{1}x+\lambda_{2}y\right)}^{c+2}}. \end{array}$$ (3)

Let $\alpha_{1}=\frac{\lambda_{1}}{b}$ and $\alpha_{2}=\frac{\lambda_{2}}{b}$, hence (3) is reduced to

$$\begin{array}{c} f_{\left(X,Y\right)}\left(x,y\right)=\frac{\alpha_{1}\alpha_{2}c\left(c+1\right)}{{\left(1+\alpha_{1}x+\alpha_{2}y\right)}^{c+2}},\;\;\;x,y,\alpha_{1},\alpha_{2},c\;>0, \end{array}$$ (4)

which is known as the *Bivariate Lomax* distribution. It can be shown that the conditional and the joint cumulative survival function of (*X*, *Y*), the marginal of *X* and the marginal of *Y* are defined as follows:

$$\begin{array}{ccc} f_{Y|X}\left(y|x\right) & = & \frac{\alpha_{2}\left(c+1\right){\left(1+\alpha_{1}x\right)}^{\left(c+1\right)}}{{\left(1+\alpha_{1}x+\alpha_{2}y\right)}^{\left(c+2\right)}},\;\;x,y,c,\alpha_{1},\alpha_{2}>0, \end{array}$$ (5)

$$\begin{array}{ccc} 1-F_{\left(X,Y\right)}\left(x,y\right) & = & {\left(1+\alpha_{1}x+\alpha_{2}y\right)}^{-c},\;\;x,y,c,\alpha_{1},\alpha_{2}>0, \end{array}$$ (6)

$$\begin{array}{ccc} f_{X}\left(x\right) & = & \frac{c\alpha_{1}}{{\left(1+\alpha_{1}x\right)}^{\left(c+1\right)}},\;\;x,c,\alpha_{1},\alpha_{2}>0, \end{array}$$ (7)

$$\begin{array}{ccc} f_{Y}\left(y\right) & = & \frac{c\alpha_{2}}{{\left(1+\alpha_{2}y\right)}^{\left(c+1\right)}},\;y,c,\alpha_{1},\alpha_{2}>0. \end{array}$$ (8)

The quantity of interest is the parameter *θ* = *P*(*X* < *Y*) which is derived as

$$\begin{array}{ccc} \theta & = & P\left(X<Y\right)=\int_{0}^{\infty }\int_{x}^{\infty }\frac{\alpha_{1}\alpha_{2}c\left(c+1\right)}{{\left(1+\alpha_{1}x+\alpha_{2}y\right)}^{c+2}}dydx\\ & = & \frac{\alpha_{1}}{\alpha_{1}+\alpha_{2}}. \end{array}$$ (9)

## 3. Classical estimation procedures

### 3.1. Maximum likelihood estimator for *θ*

Using the definition given by [19]. Let *X*_*obs*_ and *Y*_*obs*_ be the observed data such that

$$\begin{array}{ccc} X_{obs} & = & {\left(X_{1:m:n},X_{2:m:n},\ldots ,X_{m:m:n}\right)}^{T},\\ Y_{obs} & = & {\left(Y_{1:m:n},Y_{2:m:n},\ldots ,Y_{m:m:n}\right)}^{T}, \end{array}$$

and *X*_*Cen*_ and *Y*_*Cen*_ represent the censored data defined as

$$\begin{array}{ccc} \;X_{Cen} & = & {\left(X_{\left(1\right)},X_{\left(2\right)},\ldots ,X_{\left(m\right)}\right)}^{T},\\ \;Y_{Cen} & = & {\left(Y_{\left(1\right)},Y_{\left(2\right)},\ldots ,Y_{\left(m\right)}\right)}^{T}, \end{array}$$

where *X*_(*i*)_ and *Y*_(*i*)_ are 1 × *R*_*i*_ vectors with $X_{\left(i\right)}=\left(X_{i1},X_{i2},\ldots ,X_{iR_{i}}\right)$ and $Y_{\left(i\right)}=\left(Y_{i1},Y_{i2},\ldots ,Y_{iR_{i}}\right),i=1,2,\ldots ,m$. By combining (*X*_*obs*_, *Y*_*obs*_) and (*X*_*Cen*_, *Y*_*Cen*_) we get the the complete data (**X**, **Y**), where $X=\left(X_{obs}^{T},X_{Cen}^{T}\right)$ and $Y=\left(Y_{obs}^{T},Y_{Cen}^{T}\right)$. The joint density of (**X**,**Y**) is given by

$$\begin{array}{ccc} f_{\left(X,Y\right)}\left(x,y\right)\\ & = & C_{1}\overset{m}{\underset{i=1}{\Pi }}\overset{R_{i}}{\underset{k=1}{\Pi }}f_{Y|X}\left(y_{i:m:n}|x_{i:m:n}\right)f_{X}\left(x_{i:m:n}\right)f_{Y|X}\left(y_{ik}|x_{ik}\right)f_{X}\left(x_{ik}\right),\\ x_{1:m:n} & < & \ldots <x_{m:m:n},\;\;x_{i:m:n}\leq x_{ik}<\infty ,\;\;k=1,\ldots ,R_{i},\;i=1,\ldots ,m,\\ C_{1} & = & n\left(n-R_{1}-1\right)\cdots \left(n-R_{1}-R_{2}-\cdots -R_{m-1}-m+1\right). \end{array}$$ (10)

The joint density of (*X*_*obs*_,*Y*_*obs*_) can be written as

$$\begin{array}{ccc} f_{\left(X_{obs},Y_{obs}\right)}\left(x_{obs},y_{obs}\right) & = & C_{1}\overset{m}{\underset{i=1}{\Pi }}f_{Y|X}\left(y_{i:m:n}|x_{i:m:n}\right)f_{X}\left(x_{i:m:n}\right){\left[1-F_{X}\left(x_{i:m:n}\right)\right]}^{R_{i}},\\ \;x_{1:m:n} & \leq & \ldots \leq x_{m:m:n},\;\;-\infty <y_{i:m:n}<\infty . \end{array}$$ (11)

Let (*X*_*i*:*m*:*n*_, *Y*_[*i*:*m*:*n*]_), *i* = 1, 2, …, *m* be *m* pairs of independent random samples from the *Bivariate Lomax* distribution defined in (4) with progressive censoring scheme **R** = (*R*_1_, …, *R*_*m*_) where *R*_*i*_ ≥ 0, $n=m+\sum_{i=1}^{m}R_{i}$. Then, the associated likelihood function *L* and its corresponding log likelihood function *l* are given by:

$$\begin{array}{ccc} L & = & C_{1}{\left(\alpha_{1}\alpha_{2}c\left(c+1\right)\right)}^{m}\overset{m}{\underset{i=1}{\Pi }}{\left(1+\alpha_{1}x_{i:m:n}+\alpha_{2}y_{i:m:n}\right)}^{-c\left(R_{i}+1\right)-2}, \end{array}$$ (12)

and,

$$\begin{array}{ccc} l & \propto & m\;\text{ln}\;\alpha_{1}+m\;\text{ln}\;\left(\alpha_{2}\right)+m\;\text{ln}\;c\left(c+1\right)\\ & & -\left(c\left(R_{i}+1\right)+2\right)\;\text{ln}\;\left(1+\alpha_{1}x_{i:m:n}+\alpha_{2}y_{i:m:n}\right). \end{array}$$ (13)

Then, the *MLEs* of *α*_1_, *α*_2_ and *c*, denoted by ${\hat{\alpha }}_{1MLE}$, ${\hat{\alpha }}_{2MLE}$ and ${\hat{c}}_{MLE},$ respectively, are the solutions of the following loglikelihood equations

$$\begin{array}{ccc} \frac{\partial l}{\partial \alpha_{1}} & = & \frac{m}{\alpha_{1}}-\left(c\left(R_{i}+1\right)+2\right)\overset{m}{\sum_{i=1}}\frac{x_{i:m:n}}{\left(1+\alpha_{1}x_{i:m:n}+\alpha_{2}y_{i:m:n}\right)}=0, \end{array}$$ (14)

$$\begin{array}{c} \frac{\partial l}{\partial \alpha_{2}}=\frac{m}{\alpha_{2}}-\left(c\left(R_{i}+1\right)+2\right)\overset{m}{\sum_{i=1}}\frac{y_{i:m:n}}{\left(1+\alpha_{1}x_{i:m:n}+\alpha_{2}y_{i:m:n}\right)}=0, \end{array}$$ (15)

and,

$$\begin{array}{ccc} \frac{\partial l}{\partial c} & = & \frac{m\left(2c+1\right)}{c\left(c+1\right)}-\left(R_{i}+1\right)\overset{m}{\sum_{i=1}}\text{ln}\left(1+\alpha_{1}x_{i:m:n}+\alpha_{2}y_{i:m:n}\right)=0. \end{array}$$ (16)

Note that there is no explicit solution to (14), (15) and (16). Hence, numerical methods are applied to obtain the *MLEs* of *α*_1_, *α*_2_ and *c*. Once ${\hat{\alpha }}_{1MLE}$, ${\hat{\alpha }}_{2MLE}$ are obtained, the *MLE* of *θ* denoted by ${\hat{\theta }}_{MLE},$ is obtained using the invariance property of *MLEs* as

$$\begin{array}{c} {\hat{\theta }}_{MLE}=\frac{{\hat{\alpha }}_{1MLE}}{{\hat{\alpha }}_{1MLE}+{\hat{\alpha }}_{2MLE}}. \end{array}$$ (17)

### 3.2. Method of moments estimator for *θ*

Based on censoring samples, the method of moments is less discussed in the literature due to the complicated construction of the suitable moment equations. [20] used the method of moments to estimate the shape and scale parameters for the Weibull distribution under type I censoring. [20] proposed a systematic and unifiable moment estimator of the exponential distribution parameters based on type I and type II censored samples using the concept of “Mean Residual Lifetime” (*MRL*).

In the shadow of [21], we will introduce the method of moments estimator based on a progressively type II censored sample as follows:

Let *X*_1:*m*:*n*_, *X*_2:*m*:*n*_, …, *X*_*m*:*m*:*n*_ be a progressively type II censored sample from a distribution with *cdf* *F*(*x*) and censoring scheme **R** = (*R*_1_, *R*_2_, …, *R*_*m*_) and set

$$\begin{array}{c} U=\frac{1}{n}[\overset{m}{\sum_{i=1}}X_{i:m:n}+\overset{m}{\sum_{i=1}}R_{i}\left(X_{i:m:n}+MRL\left(X_{i:m:n}\right)\right)]. \end{array}$$ (18)

such that,

$$\begin{array}{c} MRL\left(t\right)=\int_{0}^{\infty }xdF_{t}\left(x\right)=\frac{1}{1-F\left(t\right)}\left(E\left(X\right)-\int_{0}^{t}\left(1-F\left(y\right)\right)dy\right), \end{array}$$ (19)

with,

$$\begin{array}{c} F_{t}\left(x\right)=P\left(X\leq t+x|X>t\right). \end{array}$$ (20)

**Theorem**: *For general lifetime distribution, the moment estimating equation is E*(*X*) = *U*, where *U* as in Eq (18).

**Proof**:

Define

$$\begin{array}{c} X_{i}^{*}=X_{i}I_{i}+\left(1-I_{i}\right)g\left(X_{i:m:n}\right), \end{array}$$ (21)

where, *g*(*X*_*i*:*m*:*n*_) = *E*(*X*_*ji*_|*X*_*ji*_ > *X*_*i*:*m*:*n*_) and *X*_*ji*_ represents the *jth* removed object after the *ith* failure and *I*_*i*_ is defined as follows

$$\begin{array}{c} I_{i}=\{\begin{array}{c} 1,\\ 0, \end{array}\begin{array}{c} \;\;\;\;\;\;\;\;\;\;\;X_{i}\in \left\{X_{1:m:n},X_{2:m:n},...,X_{m:m:n}\right\}\\ X_{i}\in \left\{X_{1i},X_{2i},...,X_{R_{i}i}\right\}. \end{array} \end{array}$$ (22)

Hence, for $X_{i}^{*}$ in Eq (21)

$$\begin{array}{c} \;X_{i}^{*}=\{\begin{array}{c} X_{i:m:n},\\ g\left(X_{1:m:n}\right),\\ g\left(X_{2:m:n}\right),\\ \vdots \\ g\left(X_{m:m:n}\right), \end{array}\begin{array}{c} \;i=1,2,\ldots ,m\\ \text{if}\;X_{i}^{*}\;\text{represents}\;\text{one}\;\text{of}\;\text{the}\;\text{removed}\;\text{objects}\;\text{after}\;\text{the}\;1^{st}\;\text{failure}\\ \text{if}\;X_{i}^{*}\;\text{represents}\;\text{one}\;\text{of}\;\text{the}\;\text{removed}\;\text{objects}\;\text{after}\;\text{the}\;2^{nd}\;\text{failure}\\ \vdots \\ \text{if}\;X_{i}^{*}\;\text{represents}\;\text{one}\;\text{of}\;\text{the}\;\text{removed}\;\text{objects}\;\text{after}\;\text{the}\;m^{th}\;\text{failure.} \end{array} \end{array}$$ (23)

Hence also,

$$\begin{array}{c} \overset{n}{\sum_{i=1}}X_{i}^{*}=\overset{m}{\sum_{i=1}}X_{i:m:n}+\overset{m}{\sum_{i=1}}R_{i}\left(g\left(X_{i:m:n}\right)\right). \end{array}$$ (24)

Considering the following relation:

$$\begin{array}{c} MRL\left(X_{i:m:n}\right)=g\left(X_{i:m:n}\right)-X_{i:m:n}, \end{array}$$ (25)

therefore, we get

$$\begin{array}{c} U=\frac{\sum_{i=1}^{n}X_{i}^{*}}{n}. \end{array}$$ (26)

We now need to show,

$$\begin{array}{c} E\left(X_{i}\right)=E\left(X_{i}^{*}\right),i\in \left\{1,2,\ldots ,n\right\}. \end{array}$$ (27)

It suffices to show that,

$$\begin{array}{c} E\left(X_{i}|I_{i}=0\right)=E\left(X_{i}^{*}|I_{i}=0\right), \end{array}$$ (28)

which implies,

$$\begin{array}{c} E\left(X_{i}|I_{i}=0,X_{i:m:n}=x\right)=E\left(X_{i}^{*}|I_{i}=0,X_{i:m:n}=x\right),\;\text{for}\;\text{every}\;x\text{.} \end{array}$$ (29)

Note that,

$$\begin{array}{c} E\;\left(X_{i}^{*}|I_{i}=0,X_{i:m:n}=x\right)=g\left(x\right), \end{array}$$ (30)

while,

$$\begin{array}{c} E\left(X_{i}|I_{i}=0,X_{i:m:n}=x\right)=E\left(X_{ji}|X_{ji}>X_{i:m:n},\;X_{i:m:n}=x\right)=g\left(x\right). \end{array}$$ (31)

Thus, we get the required result

$$\begin{array}{c} E\left(X\right)=E\left(X^{*}\right)=\frac{\sum_{i=1}^{n}X_{i}^{*}}{n}=U. \end{array}$$ (32)

Hence, the proof of Theorem 1 is complete.

Now, let (*X*_*i*:*m*:*n*_, *Y*_[*i*:*m*:*n*]_), *i* = 1, 2, …, *m* be *m* pairs of independent random samples from the *Bivariate Lomax* distribution in (4). Using Eqs (7) and (8) the *cdf*’s of *X* and *Y* are given below

$$\begin{array}{ccc} F_{X}\left(x\right) & = & 1-{\left(1+\alpha_{1}x\right)}^{-c},x,\alpha_{1},c>0\\ F_{Y}\left(y\right) & = & 1-{\left(1+\alpha_{2}y\right)}^{-c},y,\alpha_{2},c>0, \end{array}$$ (33)

and the Mean Residual Lifetime function of the univariate *Lomax* distribution is

$$\begin{array}{c} MRL\left(t\right)=\frac{1}{\alpha_{1}\left(c-1\right)}+\frac{t}{\left(c-1\right)},c>1. \end{array}$$

Using (32), the moment estimators of *α*_1_ and *α*_2_, (denoted by ${\tilde{\alpha }}_{1}$ and ${\tilde{\alpha }}_{2}$), for the univariate *Lomax* lifetime model under progressive type II censoring are obtained as follows:

$$\begin{array}{ccc} {\tilde{\alpha }}_{1} & = & \frac{m}{\left(c-1\right)\sum_{i=1}^{m}X_{i:m:n}+c\sum_{i=1}^{m}R_{i}X_{i:m:n}},\\ {\tilde{\alpha }}_{2} & = & \frac{m}{\left(c-1\right)\sum_{i=1}^{m}Y_{\left[i:m:n\right]}+c\sum_{i=1}^{m}R_{i}Y_{\left[i:m:n\right]}}. \end{array}$$

Therefore, the suggested moment estimator for *θ* can be written as

$$\begin{array}{ccc} {\hat{\theta }}_{MOM} & = & \frac{{\tilde{\alpha }}_{1}}{{\tilde{\alpha }}_{1}+{\tilde{\alpha }}_{2}}\\ & = & \frac{\left(c-1\right)\sum_{i=1}^{m}Y_{\left[i:m:n\right]}+c\sum_{i=1}^{m}R_{i}Y_{\left[i:m:n\right]}}{\left(c-1\right)\sum_{i=1}^{m}Y_{\left[i:m:n\right]}+c\sum_{i=1}^{m}R_{i}Y_{\left[i:m:n\right]}+\left(c-1\right)\sum_{i=1}^{m}X_{i:m:n}+c\sum_{i=1}^{m}R_{i}X_{i:m:n}}. \end{array}$$ (34)

Notice that for large values of *c*, Eq (34) is reduced to

$$\begin{array}{c} {\hat{\theta }}_{MOM}\approx \frac{\sum_{i=1}^{m}\left(R_{i}+1\right)Y_{\left[i:m:n\right]}}{\sum_{i=1}^{m}\left(R_{i}+1\right)\left(Y_{\left[i:m:n\right]}+X_{i:m:n}\right)}. \end{array}$$ (35)

## 4. *Bayes* estimator of *θ* under progressive type II censoring

In this section, we derive the *Bayes* estimate of *θ* based on Squared and *Linex* loss functions. A commonly used loss function is the squared error loss function (*SEL*)

$$\begin{array}{c} LL\left(\hat{\theta },\theta \right)={\left(\hat{\theta }-\theta \right)}^{2}. \end{array}$$ (36)

The *Bayes* estimate under Eq (36) is the posterior mean, given by ${\hat{\theta }}_{SEL}=E_{\pi }\theta .$ The *SEL* is widely employed in *Bayesian* inference due to its computational simplicity. $LL\left(\hat{\theta },\theta \right)$ is a symmetric loss function under which overestimation and underestimation have equal weights. However, this is not a good criterion from a practical point of view. For example [22], states that in the disaster of the space shuttle, Challenger, the management may have overestimated the average life or reliability of the solid fuel rocket booster. In estimating reliability and failure rate functions, an overestimation causes more damage than underestimation. To resolve such a situation, asymmetrical loss functions are more appropriate. [23] introduced the *Linex* loss function (Linear- Exponential) in response to the criticism of the *SEL*. The *Linex* loss function has been widely used by several authors such as [24]. The *Linex* loss function is defined as follows:

$$\begin{array}{c} LL\left(\hat{\theta },\theta \right)=\text{exp}\left(\lambda \left(\hat{\theta }-\theta \right)\right)-\lambda \left(\hat{\theta }-\theta \right)-1,\;\;\lambda \neq 0. \end{array}$$ (37)

The magnitude of λ reflects the degree of symmetry while the sign of λ reflects the direction of symmetry. [25] obtained the *Bayesian* estimator under (*Linex*) loss function by minimizing the posterior expected loss as follows:

$$\begin{array}{c} {\hat{\theta }}_{LIN}=-\frac{1}{\lambda }\;\text{ln}\;E_{\pi }\left(e^{-\lambda \theta }\right), \end{array}$$ (38)

provided that *E*_*π*_(*e*^−*λθ*^) exists and is finite. The *Linex* loss function is suitable for situations where overestimation may lead to serious consequences, and it is known for its flexibility and popularity in estimating the location parameter.

Suppose that *X* and *Y* are dependent random variables from the *Bivariate Lomax* distribution with survival function given by (6). We are interested in using the *Bayesian* method to estimate $\theta =P\left(X<Y\right)=\frac{\alpha_{1}}{\alpha_{1}+\alpha_{2}}.$ To conduct the *Bayesian* method, we need to construct prior distributions on the parameters. It is assumed that the parameters *α*_1_ and *α*_2_ have independent gamma priors with known and non-negative shape parameters *a*_1_ and *a*_2_ and the same scale parameter (without loss of generality the scale parameter = 1) hence,

$$\begin{array}{c} \alpha_{1}∼\pi_{1}\left(\alpha_{1}\right)=\frac{\alpha_{1}^{a_{1}-1}e^{-\alpha_{1}}}{\Gamma \left(a_{1}\right)}\;\;\;\;\;\;\;\;\;\;\;\;\;\;\alpha_{2}∼\pi_{2}\left(\alpha_{2}\right)=\frac{\alpha_{2}^{a_{2}-1}e^{-\alpha_{2}}}{\Gamma \left(a_{2}\right)}. \end{array}$$ (39)

Following the approach of [26], we assume a *Gamma*(*a*_1_ + *a*_2_, 1) distribution for *S* = *α*_1_ + *α*_2_ with pdf

$$\begin{array}{c} \pi \left(S\right)=\frac{S^{a_{1}+a_{2}-1}e^{-S}}{\Gamma \left(a_{1}+a_{2}\right)}. \end{array}$$ (40)

Then, given *S*, $\frac{\alpha_{1}}{S}$ has a *Beta*(*a*_1_, *a*_2_) prior as follows:

$$\begin{array}{c} \pi \left(\theta \right)=\frac{\Gamma \left(a_{1}+a_{2}\right)}{\Gamma \left(a_{1}\right)\Gamma \left(a_{2}\right)}\theta^{a_{1}-1}{\left(1-\theta \right)}^{a_{2}-1}. \end{array}$$ (41)

The joint prior of of *θ* and *S* is given by

$$\begin{array}{c} \;\pi \left(\theta ,S\right)=\frac{S^{a_{1}+a_{2}-1}e^{-S}\theta^{a_{1}-1}{\left(1-\theta \right)}^{a_{2}-1}}{\Gamma \left(a_{1}\right)\Gamma \left(a_{2}\right)}. \end{array}$$ (42)

By combining (13) and (42) the joint density function of *θ* and *S* is given by

$$\begin{array}{ccc} \;\;\;\;\;\;\;\;\;\;\;\;\pi \left(\theta ,S|\underline{x},\underline{y}\right) & = & \frac{L\;\pi \left(\theta ,S\right)}{\int_{0}^{\infty }\int_{0}^{1}L\;\pi \left(\theta ,S\right)d\theta dS}\\ & = & [c\left(c+1\right){]}^{m}S^{2m+a_{1}+a_{2}-1}e^{-S}\theta^{m+a_{1}-1}{\left(1-\theta \right)}^{m+a_{2}-1}\times \\ & & \frac{\overset{m}{\underset{i=1}{\Pi }}{\left(1+\theta S\left(x_{i:m:n}-y_{\left[i:m:n\right]}\right)+S\;y_{\left[i:m:n\right]}\right)}^{-\left(c+2\right)}\overset{m}{\underset{i=1}{\Pi }}{\left(1+\theta Sx_{i:m:n}\right)}^{-cR_{i}}}{I} \end{array}$$ (43)

where,

$$\begin{array}{ccc} I & = & \int_{0}^{\infty }\int_{0}^{1}S^{2m+a_{1}+a_{2}-1}e^{-S}\theta^{m+a_{1}-1}{\left(1-\theta \right)}^{m+a_{2}-1}\\ & & \times \overset{m}{\underset{i=1}{\Pi }}{\left(1+\theta S\left(x_{i:m:n}-y_{\left[i:m:n\right]}\right)+Sy_{\left[i:m:n\right]}\right)}^{-\left(c+2\right)}\overset{m}{\underset{i=1}{\Pi }}{\left(1+\theta \;S\;x_{i:m:n}\right)}^{-cR_{i}}d\theta dS, \end{array}$$

and (*x*_*i*:*m*:*n*_, *y*_[*i*:*m*:*n*]_) represents the *i* *th* progressively type II censored ordered pairs from *Bivariate Lomax* distribution. Therefore, the *Bayes* estimators of any function of *θ* and *S*, say *w*(*θ*, *S*), are the posterior expected values. Let *w*(*θ*, *S*) be a function of *θ* and *S*, then the expected value of *w*(*θ*, *S*) is given by

$$\begin{array}{ccc} \;\;\;\;\;\;\hat{w} & = & E_{\pi }\left(w\left(\theta ,S\right)|\underline{x},\underline{y}\right)\\ & = & \frac{\int_{0}^{\infty }\int_{0}^{1}w\left(\theta ,S\right)e^{l+\rho \left(\theta ,S\right)}d\theta dS}{\int_{0}^{\infty }\int_{0}^{1}w\left(\theta ,S\right)e^{l+\rho \left(\theta ,S\right)}d\theta dS}, \end{array}$$ (44)

where *l* = ln *L* and *ρ*(*θ*, *S*) = ln *π*(*θ*, *S*).

It can be noticed that $\hat{w}$ is in the form of a ratio of two integrals which cannot be simplified to a closed form. Hence Lindley’s approximation method is applied to obtain the *Bayes* estimator of *θ*, see [27]. Then Eq (44) is reduced to the following numerical expression:

$$\begin{array}{ccc} \;\hat{w} & = & w\left(\hat{\theta },\hat{S}\right)+0.5[\left({\hat{w}}_{\theta \theta }+2{\hat{w}}_{\theta }{\hat{\rho }}_{\theta }\right){\hat{\sigma }}_{\theta \theta }+\left({\hat{w}}_{S\theta }+2{\hat{w}}_{S}{\hat{\rho }}_{\theta }\right){\hat{\sigma }}_{S\theta }+\left({\hat{w}}_{\theta S}+2{\hat{w}}_{\theta }{\hat{\rho }}_{S}\right){\hat{\sigma }}_{\theta S}]\\ & & +0.5\left({\hat{w}}_{SS}+2{\hat{w}}_{S}{\hat{\rho }}_{S}\right){\hat{\sigma }}_{SS}+0.5\left({\hat{w}}_{\theta }{\hat{\sigma }}_{\theta \theta }+{\hat{w}}_{S}{\hat{\sigma }}_{\theta S}\right)\left({\hat{l}}_{\theta \theta \theta }{\hat{\sigma }}_{\theta \theta }+{\hat{l}}_{\theta S\theta }{\hat{\sigma }}_{\theta S}\right)\\ & & +0.5\left({\hat{w}}_{\theta }{\hat{\sigma }}_{\theta \theta }+{\hat{w}}_{S}{\hat{\sigma }}_{\theta S}\right)\left({\hat{l}}_{S\theta \theta }{\hat{\sigma }}_{S\theta }+{\hat{l}}_{SS\theta }{\hat{\sigma }}_{SS}\right)\\ & & +\left({\hat{w}}_{\theta }{\hat{\sigma }}_{S\theta }+{\hat{w}}_{S}{\hat{\sigma }}_{SS}\right)\left({\hat{l}}_{S\theta \theta }{\hat{\sigma }}_{RR}+{\hat{l}}_{\theta SS}{\hat{\sigma }}_{RS}+{\hat{l}}_{S\theta S}{\hat{\sigma }}_{S\theta }+{\hat{l}}_{SSS}{\hat{\sigma }}_{SS}\right)\}. \end{array}$$ (45)

where $\hat{\theta }$ and $\hat{S}$ are the *MLEs* of *θ* and *S* respectively and ${\hat{w}}_{\theta \theta }=\frac{\partial^{2}w\left(\theta ,S\right)}{\partial \theta^{2}}{|}_{\left(\hat{\theta },\hat{S}\right)}$, ${\hat{w}}_{\theta S}={\hat{w}}_{S\theta }=\frac{\partial^{2}w\left(\theta ,S\right)}{\partial S\partial \theta }{|}_{\left(\hat{\theta },\hat{S}\right)}$, ${\hat{w}}_{SS}=\frac{\partial^{2}w\left(\theta ,S\right)}{\partial {\;S}^{2}}{|}_{\left(\hat{\theta },\hat{S}\right)}$, ${\hat{\rho }}_{\theta }=\frac{\partial \rho \left(\theta ,S\right)}{\partial \theta }{|}_{\left(\hat{\theta },\hat{S}\right)}=\frac{a_{1}-1}{\hat{\theta }}-\frac{a_{2}-1}{\left(1-\hat{\theta }\right)}$ and ${\hat{\rho }}_{S}=\frac{\partial \rho \left(\theta ,S\right)}{\partial S}{|}_{\left(\hat{\theta },\hat{S}\right)}=\frac{a_{1}+a_{2}-1}{\hat{S}}-1$. Other expressions can be defined similarly (see the S1 Appendix).

In this paper we are interested in estimating *θ*, thus *w*(*θ*, *S*) will be considered as a function of *θ* only and *w*_*S*_ = *w*_*Sθ*_ = *w*_*θS*_ = *w*_*SS*_ = 0. Hence, Eq (45) is reduced to

$$\begin{array}{ccc} \hat{w} & = & w\left(\hat{\theta },\hat{S}\right)+0.5{\hat{w}}_{\theta \theta }{\hat{\sigma }}_{\theta \theta }+{\hat{w}}_{\theta }\left[{\hat{\sigma }}_{\theta \theta }\left({\hat{\rho }}_{\theta }+{\hat{l}}_{\theta \theta S}{\hat{\sigma }}_{\theta S}\right)+{\hat{\sigma }}_{\theta S}\left({\hat{\rho }}_{S}+{\hat{l}}_{\theta SS}{\hat{\sigma }}_{\theta S}\right)\right]\\ & & +0.5{\hat{w}}_{\theta }\left[{\hat{\sigma^{2}}}_{\theta \theta }{\hat{l}}_{\theta \theta \theta }+{\hat{\sigma }}_{\theta \theta }\left({\hat{\sigma }}_{RS}{\hat{l}}_{\theta \theta S}+{\hat{\sigma }}_{SS}{\hat{l}}_{\theta SS}\right)+{\hat{\sigma }}_{\theta S}{\hat{l}}_{SSS}{\hat{\sigma }}_{SS}\right]. \end{array}$$ (46)

### 4.1. *Bayes* estimate of *θ*

- Approximate *Bayes* estimate of *θ* under squared error loss function.

If *w*(*θ*, *S*) = *θ*, *w*_*θ*_ = 1, *w*_*θθ*_ = 0. Then Lindley’s approximation of the *Bayes* estimator ${\hat{\theta }}_{SEL}$ is

$$\begin{array}{ccc} {\hat{\theta }}_{SEL} & = & \hat{\theta }+{\hat{\sigma }}_{\theta \theta }\left({\hat{\rho }}_{\theta }+{\hat{l}}_{\theta \theta S}{\hat{\sigma }}_{\theta S}\right)+{\hat{\sigma }}_{\theta S}\left({\hat{\rho }}_{S}+{\hat{l}}_{\theta SS}{\hat{\sigma }}_{\theta S}\right)\\ & & +0.5[{\hat{\sigma^{2}}}_{\theta \theta }{\hat{l}}_{\theta \theta \theta }+{\hat{\sigma }}_{\theta \theta }\left({\hat{\sigma }}_{\theta S}{\hat{l}}_{\theta \theta S}+{\hat{\sigma }}_{SS}{\hat{l}}_{\theta SS}\right)\\ & & +{\hat{\sigma }}_{\theta S}{\hat{l}}_{SSS}{\hat{\sigma }}_{SS}]. \end{array}$$ (47)

- Approximate *Bayes* estimate of *θ* under *Linex* loss function.

If *w*(*θ*, *S*) = *e*^−λ*θ*^, *w*_*θ*_ = −λ*e*^−λ*θ*^, *w*_*θθ*_ = λ^2^ *e*^−λ*θ*^. Then

$$\begin{array}{ccc} & & E_{\pi }\left(e^{-\lambda \theta }|\underline{x},\underline{y}\right)=-0.5\lambda e^{-\lambda \hat{\theta }}\left[{\hat{\sigma^{2}}}_{\theta \theta }{\hat{l}}_{\theta \theta \theta }+{\hat{\sigma }}_{\theta \theta }\left({\hat{\sigma }}_{\theta S}{\hat{l}}_{\theta \theta S}+{\hat{\sigma }}_{SS}{\hat{l}}_{\theta SS}\right)+{\hat{\sigma }}_{\theta S}{\hat{l}}_{SSS}{\hat{\sigma }}_{SS}\right]\\ & & +e^{-\lambda \hat{\theta }}+0.5\lambda^{2}e^{-\lambda \hat{\theta }}{\hat{\sigma }}_{\theta \theta }-\lambda e^{-\lambda \hat{\theta }}[{\hat{\sigma }}_{\theta \theta }\left({\hat{\rho }}_{\theta }+{\hat{l}}_{\theta \theta S}{\hat{\sigma }}_{\theta S}\right)+{\hat{\sigma }}_{\theta S}\left({\hat{\rho }}_{S}+{\hat{l}}_{\theta SS}{\hat{\sigma }}_{\theta S}\right)], \end{array}$$

and hence, the *Bayes* estimate ${\hat{\theta }}_{LIN}$ is obtained by

$$\begin{array}{c} {\hat{\theta }}_{LIN}=-\frac{1}{\lambda }\;\text{ln}\;E_{\pi }\left(e^{-\lambda \theta }|\underline{x},\underline{y}\right). \end{array}$$ (48)

## 5. Simulation study

The purpose of the simulation study is to compare the performance of the classical estimates (*MLE* and *MOM*) and the *Bayesian* estimates based on symmetric and asymmetric loss functions using independent gamma priors for *α*_1_ and *α*_2_ as provided in Eq (39). Progressively censored samples are randomly generated from *Bivariate Lomax* as follows.

1. Values of *α*_1_ and *α*_2_ are generated from *π*_1_(*α*_1_) and *π*_2_(*α*_2_) as given in Eq (39) with specified parameters *a*_1_ and *a*_2_. The resulting values of *α*_1_ and *α*_2_ are considered to be the true values that will be used to generate a bivariate progressive type-II censored sample.
2. For given *m*, *n*, *c*, (*R*_1_, *R*_2_, …, *R*_*m*_) and and the resulted values of *α*_1_ and *α*_2_ above, we
  (a) generate *m* independent univariate *Lomax* (*α*_1_, *c*) random variables *X*_1_, *X*_2_, …, *X*_*m*_.
  (b) generate *m* progressively type II censored samples *Y*_*i*_|*X*_*i*_, *i* = 1, 2, …, *m* using the algorithm provided by Balakrishnan and Cramer (2014). Finally, (*x*_*i*:*m*:*n*_, *y*_[*i*:*m*:*n*]_), *i* = 1, 2, …, *m* is the required progressively type-II censored sample of size *m* from the *Bivariate Lomax* distribution with parameters (*α*_1_, *α*_2_, *c*)
3. The above steps are repeated 5000 times, for each of the following values of *a*_1_( = 1, 2, 3, 8, 9), *a*_2_( = 1, 2, 6, 9), *c*( = 30), λ( = −8, 8), *n* = 70, 75, 120, *m* = 25, 50, 100 and nine different censoring schemes (*R*_1_, *R*_2_, …, *R*_*m*_) as given in Table 1. For the censoring scheme we will follow [28] notations. For instance, if *n* = 15, *m* = 10, then the censoring scheme (2, 3, 0^*8^) means that after the first failure, 2 items are removed at random from the remaining 14 items, then after the second failure, 3 items are removed at random from the remaining 11 items, then the next 8 failure times are observed.
4. In each case, the classical estimators (*MLE*, and *MOM*) and the *Bayesian* estimators based on symmetric and asymmetric loss functions using independent gamma priors for *α*_1_ and *α*_2_ are computed. We obtain the *MLE*s of *α*_1_, *α*_2_ and *c* by solving the nonlinear Eqs (14), (15) and (16) using the Newton-Raphson algorithm which is implemented in *SAS*/*IML*.

**Table 1:** Censoring scheme R = (*R*_1_, *R*_2_, …, *R*_*m*_).

n	m	Scheme	
70	25	1	(45, 0^*24^)
		2	(0^*24^, 45)
		3	(0^*4^, 10, 0^*4^, 10, …, 0^*4^, 10)
75	50	4	(25, 0^*49^)
		5	(0^*49^, 25)
		6	(0^*9^, 5, 0^*9^, 5, …, 0^*9^, 5)
120	100	7	(20, 0^*79^)
		8	(0^*79^, 20)
		9	(0^*9^, 2, 0^*9^, 2, …, 0^*9^, 2)

The three criteria used for comparing all the above estimators are the Absolute Relative Bias (*ARBias*), Mean Squared Error (*MSE*) and Pitman Nearness (*PN*) probability. Suppose ${\hat{\theta }}_{i}$ is the estimate of *θ*, for the *i* *th* simulated data set, then the *ARBias*, *MSE* and *PN* are computed as follows:

(i)
$ARBias=\sum_{i=1}^{5000}\frac{\left|{\hat{\theta }}_{i}-\theta \right|}{\theta }$ ,
(ii)
$MSE=\frac{1}{5000}\sum_{i=1}^{5000}{\left({\hat{\theta }}_{i}-\theta \right)}^{2}$ ,
(iii)
$PN=P\{|{\hat{\theta }}_{i}-\theta |<|{\hat{\theta }}_{j}-\theta |\}=\frac{1}{5000}\#\{|{\hat{\theta }}_{i}-\theta |<|{\hat{\theta }}_{j}-\theta |\}$ ,

and we say that ${\hat{\theta }}_{i}$ outperforms ${\hat{\theta }}_{j}$ if *PN* > 0.5.

All the computations are performed using SAS/IML. Results are summarized in Tables 1–5 provided at the end of this section as follows.

- Table 1 shows 9 cases of different censoring schemes.
- Tables 2 and 3 present *ARBias* and *MSE* values for the proposed estimators.
- Tables 4 and 5 display the *PN* probability of the estimators of *θ* relative to each other for large *m*.

**Table 2:** Absolute relative bias (*ARBias*) and MSE (in parentheses) of $\hat{\theta }$ with theoretical value of *θ* < 0.5.

(*α*_1_, *α*_2_, θ)		(*n*, m) = (70, 25)			(*n*, *m*) = (75, 50)			(*n*, *m*) = (120, 100)		
		*Scheme* 1	*Scheme* 2	*Scheme* 3	*Scheme* 4	*Scheme* 5	*Scheme* 6	*Scheme* 7	*Scheme* 8	*Scheme* 9
(1, 9, **0.1**)	${\hat{\theta }}_{MLE}$	0.4818 (**0.0034**)	0.8916 (**0.0082**)	0.8554 (**0.0075**)	0.2464 (**0.0013**)	0.6607 (**0.0046**)	0.5206 (**0.0029**)	0.1229 (**0.0005**)	0.4382 (**0.0021**)	0.3062 (**0.0011**)
	${\hat{\theta }}_{MOM}$	0.4895 (**0.0035**)	0.5288 (**0.0036**)	0.6432 (**0.0044**)	0.2646 (**0.0014**)	0.2799 (**0.0015**)	0.3309 (**0.0017**)	0.1421 (**0.0005**)	0.1499 (**0.0007**)	0.1658 (**0.0005**)
	${\hat{\theta }}_{SEL}$	0.7388 (**0.0097**)	0.9980 (**0.0138**)	0.9433 (**0.0150**)	0.4859 (**0.0053**)	0.8087 (**0.0092**)	0.8047 (**0.0249**)	0.2997 (**0.0026**)	0.5607 (**0.0049**)	0.4917 (**0.0048**)
	${\hat{\theta }}_{LIN,\lambda =8}$	0.5751 (**0.0075**)	0.8168 (**0.0105**)	0.7020 (**0.0109**)	0.3903 (**0.0045**)	0.6732 (**0.0072**)	0.5609 (**0.0215**)	0.2595 (**0.0024**)	0.5156 (**0.0044**)	0.4185 (**0.0042**)
	${\hat{\theta }}_{LIN,\lambda =-8}$	1.7830 (**0.0680**)	1.7814 (**0.0411**)	1.7727 (**0.0511**)	1.7921 (**0.0489**)	1.7906 (**0.0548**)	1.7913 (**0.0535**)	1.8167 (**0.1076**)	1.8145 (**0.1407**)	1.8099 (**0.1344**)
(2, 6, **0.25**)	${\hat{\theta }}_{MLE}$	0.4510 (**0.0184**)	0.8723 (**0.0477**)	0.8376 (**0.0437**)	0.2243 (**0.0062**)	0.6228 (**0.0251**)	0.4835 (**0.0155**)	0.1100 (**0.0024**)	0.3978 (**0.0107**)	0.2752 (**0.0056**)
	${\hat{\theta }}_{MOM}$	0.4714 (**0.0190**)	0.4857 (**0.0202**)	0.6176 (**0.0248**)	0.2439 (**0.0063**)	0.2508 (**0.0064**)	0.3101 (**0.0077**)	0.1286 (**0.0024**)	0.1326 (**0.0028**)	0.1517 (**0.0028**)
	${\hat{\theta }}_{SEL}$	0.5423 (**0.0337**)	0.9457 (**0.0847**)	0.8691 (**0.0682**)	0.2381 (**0.0065**)	0.7017 (**0.0460**)	0.5517 (**0.0264**)	0.1176 (**0.0025**)	0.4040 (**0.0110**)	0.3272 (**0.0211**)
	${\hat{\theta }}_{LIN,\lambda =8}$	0.5190 (**0.0322**)	0.8618 (**0.0753**)	0.7892 (**0.0600**)	0.2535 (**0.0070**)	0.6543 (**0.0420**)	0.5264 (**0.0247**)	0.1277 (**0.0026**)	0.4088 (**0.0113**)	0.3162 (**0.0206**)
	${\hat{\theta }}_{LIN,\lambda =-8}$	1.3335 (**0.1195**)	1.3323 (**0.1118**)	1.3355 (**0.1149**)	1.3354 (**0.1147**)	1.3347 (**0.1136**)	1.3340 (**0.1134**)	1.3358 (**0.1135**)	1.3356 (**0.1149**)	1.3355 (**0.1141**)
(1, 2, **0.33**)	${\hat{\theta }}_{MLE}$	0.4290 (**0.0295**)	0.8590 (**0.0823**)	0.8155 (**0.0739**)	0.2097 (**0.0096**)	0.5961 (**0.0411**)	0.4532 (**0.0244**)	0.0959 (**0.0034**)	0.3671 (**0.0162**)	0.2494 (**0.0083**)
	${\hat{\theta }}_{MOM}$	0.4433 (**0.0315**)	0.4691 (**0.0334**)	0.5773 (**0.0390**)	0.2299 (**0.0112**)	0.2366 (**0.0411**)	0.2784 (**0.0121**)	0.1155 (**0.0033**)	0.1159 (**0.0035**)	0.1323 (**0.0037**)
	${\hat{\theta }}_{SEL}$	0.4444 (**0.0354**)	0.8929 (**0.1230**)	0.8198 (**0.0827**)	0.2179 (**0.0100**)	0.6065 (**0.0428**)	0.4692 (**0.0284**)	0.1015 (**0.0036**)	0.3750 (**0.0173**)	0.2572 (**0.0090**)
	${\hat{\theta }}_{LIN,\lambda =8}$	0.4581 (**0.0367**)	0.8546 (**0.1156**)	0.7972 (**0.0787**)	0.2360 (**0.0109**)	0.6091 (**0.0432**)	0.4739 (**0.0289**)	0.1129 (**0.0039**)	0.3800 (**0.0177**)	0.2652 (**0.0095**)
	${\hat{\theta }}_{LIN,\lambda =-8}$	1.2480 (**0.1864**)	1.2455 (**0.3587**)	1.248 (**0.1847**)	1.2504 (**0.1788**)	1.2480 (**0.1784**)	1.2485 (**0.1776**)	1.2517 (**0.1758**)	1.2516 (**0.1771**)	1.2514 (**0.1760**)

**Table 3:** Absolute relative bias(*ARBias*) and *MSE* (in parentheses) of $\hat{\theta }$ with theoretical value of *θ* > 0.5.

(*α*_1_, *α*_2_, θ)		(*n*, m) = (70, 25)			(*n*, m) = (75, 50)			(*n*, m) = (120, 100)		
		*Scheme* 1	*Scheme* 2	*Scheme* 3	*Scheme* 4	*Scheme* 5	*Scheme* 6	*Scheme* 7	*Scheme* 8	*Scheme* 9
(3, 1, **0.75**)	${\hat{\theta }}_{MLE}$	0.2707 (**0.0659**)	0.7155 (**0.2996**)	0.6350 (**0.2328**)	0.1048 (**0.0134**)	0.3701 (**0.0852**)	0.2448 (**0.0382**)	0.0468 (**0.0034**)	0.1846 (**0.0220**)	0.1164 (**0.0096**)
	${\hat{\theta }}_{MOM}$	0.2920 (**0.0786**)	0.2848 (**0.0643**)	0.3578 (**0.0806**)	0.1232 (**0.0200**)	0.1141 (**0.0126**)	0.1351 (**0.0148**)	0.0599 (**0.0060**)	0.0513 (**0.0033**)	0.0595 (**0.0038**)
	${\hat{\theta }}_{SEL}$	0.2309 (**0.0654**)	0.6889 (**0.2792**)	0.5885 (**0.2009**)	0.0870 (**0.0134**)	0.3555 (**0.0787**)	0.2329 (**0.0348**)	0.0388 (**0.0034**)	0.1802 (**0.0210**)	0.1136 (**0.0092**)
	${\hat{\theta }}_{LIN,\lambda =8}$	0.2653 (**0.0755**)	0.7000 (**0.2878**)	0.6042 (**0.2115**)	0.0972 (**0.0145**)	0.3685 (**0.0841**)	0.2473 (**0.0387**)	0.0430 (**0.0036**)	0.1865 (**0.0223**)	0.1196 (**0.0100**)
	${\hat{\theta }}_{LIN,\lambda =-8}$	1.1117 (**0.7383**)	1.1114 (**0.7261**)	1.1115 (**0.7019**)	1.1117 (**0.7085**)	1.1117 (**0.7044**)	1.1117 (**0.7008**)	1.1117 (**0.6998**)	1.1117 (**0.6994**)	1.1117 (**0.6986**)
(8, 2, **0.8**)	${\hat{\theta }}_{MLE}$	0.2412 (**0.0607**)	0.6707 (**0.3030**)	0.5830 (**0.2246**)	0.0913 (**0.0113**)	0.3228 (**0.0751**)	0.2089 (**0.0320**)	0.0370 (**0.0026**)	0.1538 (**0.0176**)	0.0958 (**0.0074**)
	${\hat{\theta }}_{MOM}$	0.2637 (**0.0743**)	0.2443 (**0.0554**)	0.3103 (**0.0698**)	0.1088 (**0.0177**)	0.0949 (**0.0103**)	0.1134 (**0.0116**)	0.0476 (**0.0046**)	0.0413 (**0.0025**)	0.0489 (**0.0029**)
	${\hat{\theta }}_{SEL}$	0.1742 (**0.0454**)	0.6035 (**0.2502**)	0.4717 (**0.1493**)	0.0660 (**0.0105**)	0.2869 (**0.0600**)	0.1840 (**0.0250**)	0.0291 (**0.0024**)	0.1427 (**0.0153**)	0.0900 (**0.0066**)
	${\hat{\theta }}_{LIN,\lambda =8}$	0.1940 (**0.0501**)	0.6018 (**0.2489**)	0.4363 (**0.1287**)	0.0760 (**0.0114**)	0.2967 (**0.0637**)	0.1952 (**0.0277**)	0.0325 (**0.0025**)	0.1476 (**0.0162**)	0.0944 (**0.0071**)
	${\hat{\theta }}_{LIN,\lambda =-8}$	1.1048 (**0.8089**)	1.1048 (**0.7996**)	1.1048 (**0.7888**)	1.1048 (**0.7894**)	1.1048 (**0.7897**)	1.1048 (**0.7857**)	1.1048 (**0.7842**)	1.1048 (**0.7846**)	1.1048 (**0.7838**)
(9, 1, **0.9**)	${\hat{\theta }}_{MLE}$	0.1531 (**0.0342**)	0.5309 (**0.2511**)	0.4194 (**0.1506**)	0.0512 (**0.0046**)	0.1981 (**0.0374**)	0.1200 (**0.0137**)	0.0210 (**0.0037**)	0.0853 (**0.0070**)	0.0521 (**0.0028**)
	${\hat{\theta }}_{MOM}$	0.1720 (**0.0445**)	0.1537 (**0.0295**)	0.1883 (**0.0341**)	0.0632 (**0.0079**)	0.0516 (**0.0040**)	0.0622 (**0.0045**)	0.0281 (**0.0021**)	0.0218 (**0.0009**)	0.0263 (**0.0011**)
	${\hat{\theta }}_{SEL}$	0.0445 (**0.1021**)	0.4480 (**0.1853**)	0.3259 (**0.0952**)	0.0017 (**0.0184**)	0.1693 (**0.0277**)	0.0893 (**0.0108**)	0.0047 (**0.0037**)	0.0777 (**0.0059**)	0.0434 (**0.0022**)
	${\hat{\theta }}_{LIN,\lambda =8}$	0.0883 (**0.1068**)	0.4414 (**0.1805**)	0.3213 (**0.0928**)	0.0085 (**0.0185**)	0.1745 (**0.0292**)	0.0937 (**0.0115**)	0.0068 (**0.0037**)	0.0796 (**0.0061**)	0.0447 (**0.0023**)
	${\hat{\theta }}_{LIN,\lambda =-8}$	1.0933 (**1.0614**)	1.0933 (**0.9900**)	1.0932 (**0.9729**)	1.0933 (**0.9936**)	1.0933 (**0.9710**)	1.0933 (**0.9734**)	1.0933 (**0.9692**)	1.0933 (**0.9672**)	1.0933 (**0.9669**)

**Table 4:** *PN* comparisons based on progressive type II censoring based on three types of censoring with theoretical value of *θ* < 0.5.

(*α*_1_, *α*_2_, θ)	Estimators	1*st* type	2*nd* type	3*rd* type
(1,9,**0.1**)	${\hat{\theta }}_{MLE}$ vs ${\hat{\theta }}_{MOM}$	0.681	0.037	0.084
	${\hat{\theta }}_{MLE}$ vs. ${\hat{\theta }}_{SEL}$	0.848	0.488	0.987
	${\hat{\theta }}_{MLE}$ vs ${\hat{\theta }}_{LIN}$	0.841	0.39	0.971
	${\hat{\theta }}_{M0M}$ vs ${\hat{\theta }}_{SEL}$	0.741	0.995	0.963
	${\hat{\theta }}_{MOM}$ vs ${\hat{\theta }}_{LIN}$	0.738	0.899	0.947
	${\hat{\theta }}_{SEL}$ vs ${\hat{\theta }}_{LIN}$	0.515	0.581	0.538
(2,6,**0.25**)	${\hat{\theta }}_{MLE}$ vs ${\hat{\theta }}_{MOM}$	0.681	0.033	0.081
	${\hat{\theta }}_{MLE}$ vs ${\hat{\theta }}_{SEL}$	0.539	0.690	0.511
	${\hat{\theta }}_{MLE}$ vs ${\hat{\theta }}_{LIN}$	0.664	0.813	0.679
	${\hat{\theta }}_{M0M}$ vs ${\hat{\theta }}_{SEL}$	0.344	0.969	0.929
	${\hat{\theta }}_{MOM}$ vs ${\hat{\theta }}_{LIN}$	0.405	0.974	0.939
	${\hat{\theta }}_{SEL}$ vs ${\hat{\theta }}_{LIN}$	0.789	0.987	0.972
(1,2,**0.33**)	${\hat{\theta }}_{MLE}$ vs ${\hat{\theta }}_{MOM}$	0.672	0.034	0.080
	${\hat{\theta }}_{MLE}$ vs ${\hat{\theta }}_{SEL}$	0.552	0.589	0.579
	${\hat{\theta }}_{MLE}$ vs ${\hat{\theta }}_{LIN}$	0.773	0.962	0.978
	${\hat{\theta }}_{M0M}$ vs ${\hat{\theta }}_{SEL}$	0.349	0.967	0.935
	${\hat{\theta }}_{MOM}$ vs ${\hat{\theta }}_{LIN}$	0.390	0.970	0.942
	${\hat{\theta }}_{SEL}$ vs ${\hat{\theta }}_{LIN}$	0.768	0.979	0.969

**Table 5:** *PN* comparisons based on progressive type II censoring based on three types of censoring with theoretical value of *θ* > 0.5.

(*α*_1_, *α*_2_, θ)	Estimators	1*st* type	2*nd* type	3rd type
(3,1,**0.75**)	${\hat{\theta }}_{MLE}$ vs ${\hat{\theta }}_{MOM}$	0.679	0.025	0.078
	${\hat{\theta }}_{MLE}$ vs. ${\hat{\theta }}_{SEL}$	0.271	0.029	0.041
	${\hat{\theta }}_{MLE}$ vs ${\hat{\theta }}_{LIN}$	0.769	0.971	0.974
	${\hat{\theta }}_{M0M}$ vs ${\hat{\theta }}_{SEL}$	0.353	0.972	0.919
	${\hat{\theta }}_{MOM}$ vs ${\hat{\theta }}_{LIN}$	0.392	0.978	0.929
	${\hat{\theta }}_{SEL}$ vs ${\hat{\theta }}_{LIN}$	0.755	0.999	0.981
(8,2,**0.8**)	${\hat{\theta }}_{MLE}$ vs ${\hat{\theta }}_{MOM}$	0.679	0.078	0.078
	${\hat{\theta }}_{MLE}$ vs ${\hat{\theta }}_{SEL}$	0.259	0.090	0.090
	${\hat{\theta }}_{MLE}$ vs ${\hat{\theta }}_{LIN}$	0.332	0.379	0.379
	${\hat{\theta }}_{M0M}$ vs ${\hat{\theta }}_{SEL}$	0.314	0.916	0.916
	${\hat{\theta }}_{MOM}$ vs ${\hat{\theta }}_{LIN}$	0.327	0.925	0.925
	${\hat{\theta }}_{SEL}$ vs ${\hat{\theta }}_{LIN}$	0.742	0.982	0.982
(9,1,**0.9**)	${\hat{\theta }}_{MLE}$ vs ${\hat{\theta }}_{MOM}$	0.674	0.033	0.078
	${\hat{\theta }}_{MLE}$ vs ${\hat{\theta }}_{SEL}$	0.316	0.043	0.035
	${\hat{\theta }}_{MLE}$ vs ${\hat{\theta }}_{LIN}$	0.312	0.103	0.034
	${\hat{\theta }}_{M0M}$ vs ${\hat{\theta }}_{SEL}$	0.322	0.954	0.884
	${\hat{\theta }}_{MOM}$ vs ${\hat{\theta }}_{LIN}$	0.335	0.956	0.893
	${\hat{\theta }}_{SEL}$ vs ${\hat{\theta }}_{LIN}$	0.754	0.983	0.951

### 5.1. Results of the simulation

Throughout this subsection, we will refer to the schemes in which (*n* − *m*) items are removed at the time of the first failure by 1*st* type censoring, namely schemes 1, 4, and 7. We will refer to schemes 2, 5, and 8 where (*n* − *m*) items are removed at the time of the *m* *th* failure by the 2*nd* type censoring. Any schemes that come in between these two extremes will be called 3*rd* type censoring, namely schemes 3, 6, and 9 (see Table 1).

A summary of the results is provided below.

- From Tables 2 and 3, it is observed that the estimates obtained based on large sample sizes have smaller *ARBias* and *MSEs* as expected.
- Comparing different censoring schemes and for different theoretical values of *θ*, we noticed that the *Bayes* estimates yield similar *MSEs*. These *MSEs* improve significantly as the effective size (*m*) increases.
- It is interesting to note that when the censored data are of the 1*st* type and the theoretical value of *θ* < 0.5, ${\hat{\theta }}_{MLE}$ outperforms the *Bayes* estimates as well as ${\hat{\theta }}_{MOM},$ in terms of *MSE* and *ARBias* values.
- On the other hand, when the censored data are of the 1*st* type and the theoretical value of *θ* > 0.5, the ${\hat{\theta }}_{SEL}$ performs steadily better than the classical estimators as well as ${\hat{\theta }}_{LIN}$ in terms of *ARBias* and *MSE* values, except when *θ* = 0.9, ${\hat{\theta }}_{MOM}$ has slightly smaller *MSEs* than ${\hat{\theta }}_{SEL}$. However, as *m* increases, ${\hat{\theta }}_{MLE}$ and ${\hat{\theta }}_{LIN,\lambda =8}$ compete quite well with ${\hat{\theta }}_{SEL}$ as seen from the *MSE* values.
- It is worth mentioning that the *Linex* loss function with λ = −8 tends to produce higher *ARBias* and *MSEs*. Moreover, λ = 8 is a preferred choice for the *Linex* case. In general, as *m* and *θ* increase, *MSE* tends to decrease.
- We further observed that the method of moments estimators show superior performance compared to the *MLE* and *Bayes* estimates. In fact, ${\hat{\theta }}_{MOM}$ produces smaller *MSEs* and smaller *ARBias* as well as higher *PN* values (see Tables 4 and 5) when censored data are of the 2*nd* and 3*rd* types.
- In Tables 4 and 5, we have presented the *PN* values for all estimates based on 3 types of censoring; we can clearly see that
  -
${\hat{\theta }}_{MOM}$ outperforms *MLE* and *Bayes* estimates when data are of the 2*nd* and 3*rd* types.
  -
${\hat{\theta }}_{SEL}$ outperforms all other estimates when data are of the 1*st* type and *θ* > 0.5.
  -
${\hat{\theta }}_{MLE}$ outperforms all other estimates when data are of the 1*st* type and *θ* < 0.5.
  - It is worth mentioning that ${\hat{\theta }}_{MLE}$ is always superior to ${\hat{\theta }}_{MOM}$ for all values of *θ*, when censored data are of the 1*st* type.

## 6. Real life data

To illustrate the proposed estimators of *θ* under a *Bivariate Lomax* distribution, we used the American Football League data from the matches on three consecutive weekends in 1986. It was first published in the ‘Washington Post’ and was proposed by [29]. The validity of the exponential model is checked using Kolmogrov-Smirnov (K-S), Anderson-Darling (A-D), and Chi-square tests. In this bivariate data set (*X*, *Y*), the variable *X* represents the game time to the first points scored by kicking the ball between goal posts, while the variable *Y* represents the game time by moving the ball into the end zone. The times are given in minutes and seconds and are reported in Table 6.

**Table 6:** American Football league data.

*X*	*Y*	*X*	*Y*	*X*	*Y*
2.05	3.98	5.78	25.98	10.40	14.25
9.05	9.05	13.80	49.75	2.98	2.98
0.85	0.85	7.25	7.25	3.88	6.43
3.43	3.43	4.25	4.25	0.75	7.75
7.78	7.78	1.65	1.65	11.63	17.37
10.57	14.28	6.42	15.08	1.38	1.38
7.05	7.05	4.22	9.48	10.35	10.35
2.58	2.58	15.53	15.53	12.13	12.13
7.23	9.68	2.90	2.90	14.58	14.58
6.85	34.58	7.02	7.02	11.82	11.82
32.45	42.35	6.42	6.42	5.52	11.27
8.53	14.57	8.98	8.98	19.65	10.70
31.13	49.88	10.15	10.15	17.83	17.83
14.58	20.57	8.87	8.87	10.85	30.07

**Fig 1:**
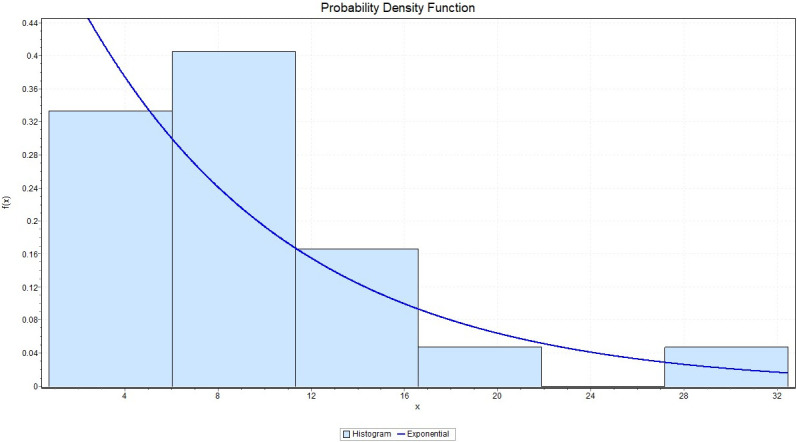
The histogram of the data set and its fitted density function to X.

**Fig 2:**
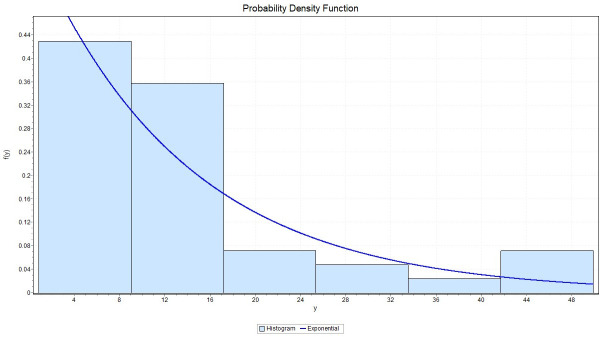
The histogram of the data set and its fitted density function to Y.

**Fig 3:**
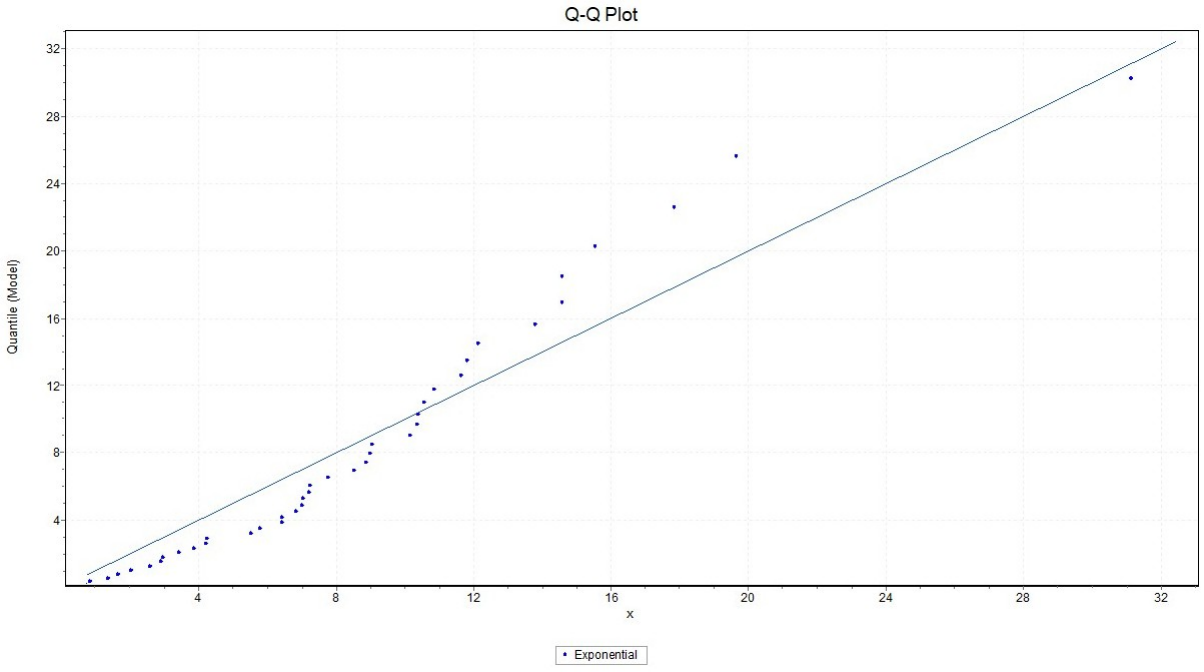
Plot of the empirical quantile of exponential distribution fitted to the X data set.

**Fig 4:**
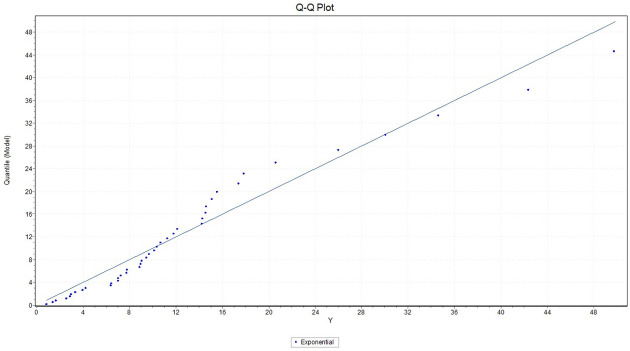
Plot of the empirical quantile of exponential distribution fitted to the Y data set.

We fit the exponential distribution for *X* with failure rate 0.1102, we observed that K-S = 0.17379 with *P*_*value*_ = 0.14023, A-D = 1.7151 and chi-square = 2.9102 with a corresponding *P*_*value*_ = 0.40569. While for *Y* we fit the exponential distribution with failure rate 0.07449, and we observed that K-S = 0.14201 with *P*_*value*_ = 0.3332, A-D = 0.80191 and chi-square = 3.0078 with a corresponding *P*_*value*_ = 0.55652. This indicates that the Exponential model provides a good fit to the above two data sets. Figs (1) and (2) give the histograms of the two data-sets and the plots of the fitted densities. The QQ plots for *X* and *Y* given in Figs (3) and (4) suggest that Exponential is very suitable for these data sets.

Next, we construct the *Bivariate Lomax* distribution to the data by using Eqs (1)–(3) and *η*λ_1_ = 0.1102, *η*λ_2_ = 0.07449, where *η* is any positive value. For simplicity of calculations, we choose *η* = 1. We fit the *Bivariate Lomax* distribution to the data and obtained the *MLE* of *θ* as $\hat{\theta }=0.5966.$ We modify the data to make it progressively censored data with three different censoring schemes given in Tables 7 and 8.

**Table 7:** Censoring schemes for American Football league.

case	censoring scheme
1	{14, 0^*27^}
2	{0^*27^, 14}
3	{1, 0, 1, 0, …, 1, 0, 1, 0}

**Table 8:** Censored American Football league.

n	m	scheme	censored data
42	28	1	(2.05,3.98), (13.8,3.98), (7.25,7.25), (4.25,4.25), (1.65,1.65) (6.42,15.08), (4.22,9.48), (15.53,15.53), (2.90,2.90), (7.02,7.02) (6.42,6.42), (8.98,8.98), (10.15,10.15), (8.87,8.87), (10.40,14.25) (2.98,2.98),(3.88,6.43), (0.75,0.75), (11.63,17.37), (1.38,1.38) (10.35,10.35),(12.13,12.13),(14.58,14.58), (11.82,11.82), (5.52,11.27) (19.65,10.70), (17.83,17.83), (10.85,38.07)
42	28	2	(2.05,3.98), (9.05,9.05), (0.85,0.85), (3.43,3.43), (7.78,7.78) (10.57,14.28), (7.05,7.05),(2.58,2.58), (7.23,9.68), (6.85,34.58) (32.45,42.35), (8.53,14.57), (31.13,49.88), (14.58,20.57), (5.78,25.98) (13.80,49.75), (7.25,7.25), (4.25,4.25), (1.65,1.65), (6.42,15.08) (4.22,9.48), (15.53,15.53),(2.90,2.90), (7.02,7.02), (6.42,6.42) (8.98,8.98), (10.15,10.15), (8.87,8.87)
42	28	3	(2.05,3.98), (0.85,0.85), (3.43,3.43), (10.57,14.28), (7.05,7.05) (7.23,9.68), (6.85,34.58), (8.53,14.57), (31.13,49.88), (5.78,25.98) (13.8,49.75), (4.25,4.25), (1.65,1.65), (4.22,9.48), (15.53,15.53) (7.02,7.02), (6.42,6.42), (10.15,10.15), (8.87,8.87), (2.98,2.98) (3.88,6.43), (11.63,17.37), (1.38,1.38), (12.13,12.13), (14.58,14.58) (5.52,11.27), (19.65,10.7), (10.85,38.07)

The parametric bootstrap percentile method is used to compute the bootstrap estimates (BootEst) and their corresponding standard error (StdErr). A 95% confidence interval is calculated and reported in terms of (LowerCI, UpperCI). The output of the bootstrap analysis is summarized in Table 9.

**Table 9:** Bootstrap results: Bootstrap estimates (BootEst), standard error (StdErr) and 95% confidence interval (LowerCI, UpperCI) for progressively censored data over 1000 resamples.

		Estimates	BootEst	SdtErr	LowerCI	UpperCI
case 1	${\hat{\theta }}_{PMLE}$	0.4556	0.4800	0.0527	0.3787	0.5829
	${\hat{\theta }}_{PMOM}$	0.7266	0.5665	0.0637	0.4455	0.7380
	${\hat{\theta }}_{PSEL}$	0.4359	0.4602	0.0529	0.3616	0.5643
	${\hat{\theta }}_{PLIN}$	0.4339	0.4582	0.0529	0.3597	0.5624
case 2	${\hat{\theta }}_{PMLE}$	0.5096	0.5235	0.0449	0.4341	0.6081
	${\hat{\theta }}_{PMOM}$	0.5411	0.5997	0.0768	0.5099	0.7861
	${\hat{\theta }}_{PSEL}$	0.4885	0.5025	0.0464	0.4117	0.5904
	${\hat{\theta }}_{PLIN}$	0.4864	0.5005	0.0464	0.4097	0.5885
case 3	${\hat{\theta }}_{PMLE}$	0.4993	0.5368	0.0450	0.4883	0.5951
	${\hat{\theta }}_{PMOM}$	0.741	0.5766	0.0396	0.5194	0.6217
	${\hat{\theta }}_{PSEL}$	0.4790	0.5184	0.0456	0.4701	0.5773
	${\hat{\theta }}_{PLIN}$	0.4770	0.5165	0.0456	0.4682	0.5755

According to the different proposed censoring schemes, it can be noticed that the bootstrap estimates are close to the true value of *θ* under each case of the proposed censoring schemes, especially for ${\hat{\theta }}_{MLE}$, ${\hat{\theta }}_{SEL}$ and ${\hat{\theta }}_{LIN}$. Also, scheme 3 provides the smallest standard error and the shortest confidence intervals.

## 7. Conclusions and recommendations

The importance of drawing inferences about *θ* = *P*(*X* < *Y*) arises naturally in many disciplines, including but not limited to: quality control, reliability, psychology, and medical applications, particularly in screening tests to verify diseased from non-diseased patients using the area under the receiver operating characteristic (*ROC*) curves, where *θ* is interpreted as an index of accuracy (Samawi et al. 2016). Consequently, it is obvious that studying reliability measures of the type *θ* = *P*(*X* < *Y*) is essential to several areas of scientific research. Therefore, it is of interest to find a reliable estimate of *θ*.

Progressive censoring has received a great deal of attention from many researchers, and this is due to its advantages in reducing the cost and time of the tests and to saving some active items for other tests.

In this paper, the authors have considered the estimation of the stress-strength reliability when the stress and the strength are dependent random variables following a *Bivariate Lomax* distribution under progressively type II censoring.The authors have derived the *MLE*, the method of moments, and the *Bayes* estimators for *θ*. Squared error and *Linex* loss functions are used for deriving the *Bayes* estimators assuming suitable priors on the unknown parameters based on progressive type II censored samples from a *Bivariate Lomax.* The *Bayes* estimates of *θ* do not have explicit form. Therefore, the authors have used Lindley’s approximation method.

An intensive simulation study has been conducted to evaluate the performance of the proposed estimators. From the simulation study, it has been noticed that progressive censoring of the 2*nd* and 3*rd* type could provide the method of moments estimator with significantly smaller biases (*ARBias*) and *MSEs* for estimating *θ*. The 2*nd* type censoring scheme, namely *R* = (0, 0, …, 0, *n*−*m*) is easy to operate in practice; besides it has the merit of good performance. On the other hand, the progressive censoring of the 1*st* type provides ${\hat{\theta }}_{MLE}$ with the smallest bias and smallest *MSEs* only when the theoretical value of *θ* is less than 0.5. However, when the theoretical value of *θ* is greater than 0.5, ${\hat{\theta }}_{SEL}$ outperforms the other estimators in most cases. It is observed that, based on *ARBias*, *MSEs* and *PN* values, the *Bayes* estimates have similar performances. In addition, by increasing the effective size *m*, expected improvements are observed in the performance of all estimators.

In practice, for the system to work, we need to consider the case when *θ* > 0.5, thus, we recommend using the method of moments if we are using 2*nd* and 3*rd* type censoring and ${\hat{\theta }}_{SEL}$ if we are using 1*st* type censoring.

## Supporting information

S1 Appendix (PDF)

S1 Data (PDF)
