# Supplementary material for: Inference on P(X < Y) in Bivariate Lomax model based on progressive type II censoring
Source: PLoS One. 2022 May 12;17(5):e0267981. doi: 10.1371/journal.pone.0267981 (PMC9098055; doi:10.1371/journal.pone.0267981)
Supplement: S1 Appendix — (PDF) [file pone.0267981.s001.pdf]

## Appendix A

For notation simplicity, we used $X_i$ and $Y_i$ instead of $X_{i:m:n}$ and $Y_{i:m:n}$ respectively. The following equations give the entries for Lindey's approximation:

$$\hat{\sigma} = \begin{bmatrix} \hat{\sigma}_{\theta\theta} & \hat{\sigma}_{\theta S} \\ \hat{\sigma}_{S\theta} & \hat{\sigma}_{SS} \end{bmatrix}^{-1} = \begin{bmatrix} -\frac{\partial^2 l}{\partial \theta^2}|_{\hat{\theta},\hat{S}} & -\frac{\partial^2 l}{\partial \theta \partial S}|_{\hat{\theta},\hat{S}} \\ -\frac{\partial^2 l}{\partial S \partial \theta}|_{\hat{\theta},\hat{S}} & -\frac{\partial^2 l}{\partial S^2}|_{\hat{\theta},\hat{S}} \end{bmatrix}^{-1}$$

$$\begin{aligned}
\hat{l}_{RR} &= \tfrac{\partial^2 l}{\partial \theta^2}|_{\hat{\theta},\hat{S}} &&= -\tfrac{m}{(1-\theta)^2} - \tfrac{m}{\theta^2} + (2+c)\sum_{j=1}^{m}(\tfrac{S(x_j-y_j)}{(1+RS(x_j-y_j)+Sy_j)})^2 \\
& && +\sum_{j=1}^{m}\tfrac{S^2R_jx_j^2}{(1+RSx_j)^2}, \\
\hat{l}_{RR} &= \tfrac{\partial^2 l}{\partial \theta^2}|_{\hat{\theta},\hat{S}} &&= -\tfrac{m}{(1-\theta)^2} - \tfrac{m}{\theta^2} + (2+c)\sum_{j=1}^{m}(\tfrac{S(x_j-y_j)}{(1+RS(x_j-y_j)+Sy_j)})^2 \\
& && +\sum_{j=1}^{m}\tfrac{S^2R_jx_j^2}{(1+RSx_j)^2},
\end{aligned}$$

$$\begin{aligned}
\hat{l}_{RRR} &= \tfrac{\partial^3 l}{\partial \theta^3}|_{\hat{\theta},\hat{S}} &&= -\tfrac{2m}{(1-\theta)^3} + \tfrac{2m}{\theta^3} - 2(2+c)\sum_{j=1}^{m}(\tfrac{S(x_j-y_j)}{(1+RS(x_j-y_j)+Sy_j)})^3 \\
& && -2c\sum_{j=1}^{m}\tfrac{S^3R_jx_j^3}{(1+RSx_j)^3}, \\
\hat{l}_{RS} \equiv \hat{l}_{SR} &= \tfrac{\partial^2 l}{\partial \theta \partial S}|_{\hat{\theta},\hat{S}} &&= (2+c)\sum_{j=1}^{m}[\tfrac{S(x_j-y_j)(\theta(x_j-y_j)+y_j)}{(1+RS(x_j-y_j)+Sy_j)^2} - \tfrac{x_j-y_j}{1+RS(x_j-y_j)+Sy_j}] \\
& && -c\sum_{j=1}^{m}[-\tfrac{RSk_jx_j^2}{(1+RSx_j)^2} + \tfrac{R_jx_j^2}{(1+RSx_j)}],
\end{aligned}$$

$$\begin{aligned}
\hat{l}_{SS} &= \tfrac{\partial^2 l}{\partial\ S^2}|_{\hat{\theta},\hat{S}} &&= -\tfrac{2m}{S^2} + (2+c)\sum_{j=1}^{m}(\tfrac{\theta(x_j-y_j)+y_j}{1++RS(x_j-y_j)+Sy_j})^2 \\
& && +c\sum_{j=1}^{m}\tfrac{\theta^2R_jx_j^2}{(1+RSx_j)^2}, \\
\hat{l}_{SSS} &= \tfrac{\partial^3 l}{\partial\ S^3}|_{\hat{\theta},\hat{S}} &&= \tfrac{4m}{S^3} - 2(2+c)\sum_{j=1}^{m}(\tfrac{\theta(x_j-y_j)+y_j}{1+RS(x_j-y_j)+Sy_j})^3 \\
& && -2c\sum_{j=1}^{m}\tfrac{\theta^3R_jx_j^3}{(1+RSx_j)^3}, \\
\hat{l}_{RSS} &= \tfrac{\partial^3 l}{\partial \theta \partial\ S^2}|_{\hat{\theta},\hat{S}} &&= -2(2+c)\sum_{j=1}^{m}[\tfrac{S(x_j-y_j)(\theta(x_j-y_j)+y_j)^2}{(1+RS(x_j-y_j)+Sy_j)^3} - \tfrac{(x_j-y_j)(\theta(x_j-y_j)+y_j)}{(1+RS(x_j-y_j)+Sy_j)^2}] \\
& && -2c\sum_{j=1}^{m}[-\tfrac{\theta^2Sk_jx_j^3}{(1+RSx_j)^3} + \tfrac{Rk_jx_j^2}{(1+RSx_j)^2}],
\end{aligned}$$

$$\begin{aligned}
\hat{l}_{RRS} &= \tfrac{\partial^3 l}{\partial \theta^2 \partial S}|_{\hat{\theta},\hat{S}} && -2(2+c)\sum_{j=1}^{m}[\tfrac{S^2(x_j-y_j)^2(\theta(x_j-y_j)+y_j)}{(1+RS(x_j-y_j)+Sy_j)^3} - \tfrac{S(x_j-y_j)^2}{(1+RS(x_j-y_j)+Sy_j)^2}] \\
& && -2c\sum_{j=1}^{m}[-\tfrac{RS^2R_jx_j^3}{(1+RSx_j)^3} + \tfrac{Sk_jx_j^2}{(1+RSx_j)^2}], \\
\hat{l}_{\theta\theta} &= \tfrac{\partial^2 l}{\partial\ \theta^2}|_{\hat{\theta},\hat{S}} &&= -\tfrac{m}{(1-\ \theta)^2} - \tfrac{m}{\theta^2} + (2+c)\sum_{j=1}^{m}(\tfrac{S(x_j-y_j)}{(1+\theta S(x_j-y_j)+Sy_j)})^2 \\
& && +\sum_{j=1}^{m}\tfrac{S^2R_jx_j^2}{(1+\theta Sx_j)^2},
\end{aligned}$$

$$
\begin{aligned}
\hat{l}_{\theta\theta\theta} &= \frac{\partial^3 l}{\partial\ \theta^3}|_{\hat{\theta},\hat{S}} &&= -\frac{2m}{(1-\ \theta)^3} + \frac{2m}{\theta^3} - 2(2+c)\sum_{j=1}^{m}(\frac{S(x_j-y_j)}{(1+\theta S(x_j-y_j)+Sy_j)})^3 \\
& && -2c\sum_{j=1}^{m}\frac{S^3R_jx_j^3}{(1+\theta Sx_j)^3}, \\
\hat{l}_{\theta S} \equiv \hat{l}_{S\theta} &= \frac{\partial^2 l}{\partial\theta\partial S}|_{\hat{\theta},\hat{S}} &&= (2+c)\sum_{j=1}^{m}[\frac{S(x_j-y_j)(\theta(x_j-y_j)+y_j)}{(1+\theta S(x_j-y_j)+Sy_j)^2} - \frac{x_j-y_j}{1+\theta S(x_j-y_j)+Sy_j}] \\
& && -c\sum_{j=1}^{m}[-\frac{\theta SR_jx_j^2}{(1+\theta Sx_j)^2} + \frac{R_jx_j^2}{(1+\theta Sx_j)}],
\end{aligned}
$$

$$
\begin{aligned}
\hat{l}_{SS} &= \frac{\partial^2 l}{\partial\ S^2}|_{\hat{\theta},\hat{S}} &&= -\frac{2m}{S^2} + (2+c)\sum_{j=1}^{m}(\frac{\theta(x_j-y_j)+y_j}{1++\theta S(x_j-y_j)+Sy_j})^2 \\
& && +c\sum_{j=1}^{m}\frac{\theta^2R_jx_j^2}{(1+\theta Sx_j)^2}, \\
\hat{l}_{SSS} &= \frac{\partial^3 l}{\partial\ S^3}|_{\hat{\theta},\hat{S}} &&= \frac{4m}{S^3} - 2(2+c)\sum_{j=1}^{m}(\frac{\theta(x_j-y_j)+y_j}{1+\theta S(x_j-y_j)+Sy_j})^3 \\
& && -2c\sum_{j=1}^{m}\frac{\theta^3R_jx_j^3}{(1+\theta Sx_j)^3}, \\
\hat{l}_{\theta SS} &= \frac{\partial^3 l}{\partial R\partial\ S^2}|_{\hat{\theta},\hat{S}} &&= -2(2+c)\sum_{j=1}^{m}[\frac{S(x_j-y_j)(\theta(x_j-y_j)+y_j)^2}{(1+\theta S(x_j-y_j)+Sy_j)^3} - \frac{(x_j-y_j)(\theta(x_j-y_j)+y_j)}{(1+\theta S(x_j-y_j)+Sy_j)^2}] \\
& && -2c\sum_{j=1}^{m}[-\frac{\theta^2SR_jx_j^3}{(1+\theta Sx_j)^3} + \frac{\theta R_jx_j^2}{(1+\theta Sx_j)^2}], \\
\hat{l}_{\theta\theta S} &= \frac{\partial^3 l}{\partial\ \theta^2\partial S}|_{\hat{\theta},\hat{S}} && -2(2+c)\sum_{j=1}^{m}[\frac{S^2(x_j-y_j)^2(\theta(x_j-y_j)+y_j)}{(1+\theta S(x_j-y_j)+Sy_j)^3} - \frac{S(x_j-y_j)^2}{(1+\theta S(x_j-y_j)+Sy_j)^2}] \\
& && -2c\sum_{j=1}^{m}[-\frac{\theta S^2R_jx_j^3}{(1+\theta Sx_j)^3} + \frac{SR_jx_j^2}{(1+\theta Sx_j)^2}].
\end{aligned}
$$

## Appendix B

```
Do i=1 to m;
Ww[i]=Uniform(seed1);
X[i]=( (1-Ww[i])**(-1/c) -1 )/alpha1;
End;
DO i=1 to m;
sum=0;
do j=m-i+1 to m;
sum=(R[j]+sum);
end;
Ee[i]=1/(i+sum);
Vv[i]=Ww[i]**Ee[i];
END;
DO g=1 to m;
prod=1;
endloop=m-g+1 ;
DO k1=m to endloop by -1;
prod=Vv*prod;
END;
U2[g]=1-(prod);
Y[g]= ( (1-U2[g])**(-1/(c+1)) * (1+alpha1*X[g]) -1-alpha1*X[g] )/ alpha2;
END;
call NLPQN(rc, paramret, LogLik, x0, opt, con,tc);
alpha1mle=paramret[1];
alpha2mle=paramret[2];
cmle=paramret[3];
gammap_mle = alpha1mle/(alpha1mle + alpha2mle);
sp_mle = (alpha1mle + alpha2mle);
```
