# Supplementary material for: Inference on P(X < Y) in Bivariate Lomax model based on progressive type II censoring
Source: PLoS One. 2022 May 12;17(5):e0267981. doi: 10.1371/journal.pone.0267981 (PMC9098055; doi:10.1371/journal.pone.0267981)
Supplement: S1 Data — (PDF) [file pone.0267981.s002.pdf]

This data was first published in the 'Washington Post'and was proposed by Csörgő and Welseh (1989).

Csörgő, S. and Welsh, A. H. (1989), Testing for exponential and Marshall Olkin distributions. *Journal of Statistical Planning and Inference*, 23(3),287-300.

Table 1: American Football League Data.

X	Y	X	Y	X	Y
2.05	3.98	5.78	25.98	10.40	14.25
9.05	9.05	13.80	49.75	2.98	2.98
0.85	0.85	7.25	7.25	3.88	6.43
3.43	3.43	4.25	4.25	0.75	7.75
7.78	7.78	1.65	1.65	11.63	17.37
10.57	14.28	6.42	15.08	1.38	1.38
7.05	7.05	4.22	9.48	10.35	10.35
2.58	2.58	15.53	15.53	12.13	12.13
7.23	9.68	2.90	2.90	14.58	14.58
6.85	34.58	7.02	7.02	11.82	11.82
32.45	42.35	6.42	6.42	5.52	11.27
8.53	14.57	8.98	8.98	19.65	10.70
31.13	49.88	10.15	10.15	17.83	17.83
14.58	20.57	8.87	8.87	10.85	30.07
